# Antisense RNA regulates glutamine synthetase in a heterocyst-forming cyanobacterium

**DOI:** 10.1093/plphys/kiae263

**Published:** 2024-05-06

**Authors:** Isidro Álvarez-Escribano, Belén Suárez-Murillo, Manuel Brenes-Álvarez, Agustín Vioque, Alicia M Muro-Pastor

**Affiliations:** Instituto de Bioquímica Vegetal y Fotosíntesis, Consejo Superior de Investigaciones Científicas and Universidad de Sevilla, 41092 Sevilla, Spain; Instituto de Bioquímica Vegetal y Fotosíntesis, Consejo Superior de Investigaciones Científicas and Universidad de Sevilla, 41092 Sevilla, Spain; Genetics and Experimental Bioinformatics, Faculty of Biology, University of Freiburg, 79104 Freiburg, Germany; Instituto de Bioquímica Vegetal y Fotosíntesis, Consejo Superior de Investigaciones Científicas and Universidad de Sevilla, 41092 Sevilla, Spain; Instituto de Bioquímica Vegetal y Fotosíntesis, Consejo Superior de Investigaciones Científicas and Universidad de Sevilla, 41092 Sevilla, Spain

## Abstract

Glutamine synthetase (GS) is a key enzyme involved in nitrogen assimilation and the maintenance of C/N balance, and it is strictly regulated in all bacteria. In cyanobacteria, GS expression is controlled by nitrogen control A (NtcA) transcription factor, which operates global nitrogen regulation in these photosynthetic organisms. Furthermore, posttranslational regulation of GS is operated by protein–protein interaction with GS inactivating factors (IFs). In this study, we describe an additional regulatory mechanism involving an antisense RNA. In *Nostoc* sp. PCC 7120, the *gifA* gene (encoding GS inactivating factor IF7) is transcribed downstream of the GS (*glnA*) gene, from the opposite strand, and the *gifA* mRNA extends into the *glnA* coding sequence in antisense orientation. Therefore, the dual RNA transcript that encodes *gifA* constitutes two functional regions: a 5′ protein-coding region, encoding IF7, and a 3′ untranslated region that acts as an antisense to *glnA*. By increasing the levels of such antisense RNA either in *cis* or in *trans*, we demonstrate that the amount of GS activity can be modulated by the presence of the antisense RNA. The tail-to-tail disposition of the *glnA* and *gifA* genes observed in many cyanobacterial strains from the Nostocales clade suggests the prevalence of such antisense RNA-mediated regulation of GS in this group of cyanobacteria.

## Teaser

The extended mRNA from an adjacent gene represses the expression of the glutamine synthetase gene through an antisense mechanism in a cyanobacterium.

## Introduction

Glutamine synthetase (GS) is one of the oldest existing and functioning enzymes (Kumada et al. 1993). GS is conserved in all domains of life, and its expression and activity are exquisitely regulated. Several regulatory mechanisms have been reported in bacteria. Enterobacterial GS, the best investigated case, is subjected to feedback inhibition by several products and posttranslationally regulated by reversible adenylylation (Kingdon et al. 1967). In contrast to other prokaryotes, cyanobacterial GS has a fundamentally different ammonium-dependent inactivating mechanism involving reversible interaction with small protein factors (Bolay et al. 2018). In addition to cumulative feedback inhibition by several metabolites (Flores and Herrero 1994), cyanobacterial GS activity is strictly regulated by nitrogen availability. The GS (*glnA*) gene, which encodes GS, is regulated at the transcriptional level by nitrogen control A (NtcA), the global nitrogen regulator. NtcA activates the transcription of *glnA* under nitrogen limitation (Herrero et al. 2001), and the activity of cyanobacterial GS is regulated at the posttranslational level by protein–protein interactions with 1 (in the case of *Nostoc* sp. PCC 7120) or 2 (in the case of *Synechocystis* sp. PCC 6803) GS inactivating factors (IFs). These factors are encoded by the GS IF A (*gifA*) and B (*gifB*) genes and named IF7 and IF17, respectively, in the case of strain PCC 6803 (García-Domínguez et al. 1999), or IF7A in the case of strain PCC 7120 (Galmozzi et al. 2010). Transcription of the *gif* genes is also regulated by NtcA, but their expression is repressed upon nitrogen limitation and derepressed upon addition of ammonium (García-Domínguez et al. 2000; Galmozzi et al. 2010). Therefore, the accumulation of *glnA* and *gifA*/*gifB* mRNAs takes place in opposite ways in response to nitrogen availability. Finally, posttranscriptional regulation by NsiR4, a nitrogen-regulated small RNA involved in CO_2_ assimilation in *Nostoc* sp. PCC 7120 (Brenes-Álvarez et al. 2021), has also been described to affect IF7 translation/accumulation in *Synechocysti*s sp. PCC 6803 (Klähn et al. 2015), while translation of IF17 in *Synechocysti*s sp. PCC 6803 is modulated by a glutamine riboswitch (Klähn et al. 2018).

Antisense transcription is consistently observed in bacterial transcriptomes (Georg and Hess 2018), and accumulating evidence suggests regulatory consequences on gene expression (Toledo-Arana and Lasa 2020). The recent definition of the transcriptome of the heterocyst-forming cyanobacterium *Nostoc* (*Anabaena*) sp. PCC 7120 reveals that antisense transcription is also prevalent in this organism, in which about 65% of the transcriptional units contain regions in antisense orientation to other transcripts (Brenes-Álvarez et al. 2023). Overlapping transcripts are commonly produced in the case of adjacent genes that are transcribed in a head-to-head or tail-to-tail disposition. This observation raises the possibility that specific physiological conditions regulating one of the overlapping partners may have consequences for the expression of an adjacent gene transcribed in the opposite direction. If two overlapping transcriptional units are regulated in response to different environmental inputs, an antisense-based mechanism would integrate those different signals beyond the effects exerted at the transcriptional level on each of the genes involved.

In *Nostoc* sp. PCC 7120 the transcriptional units covering the *glnA* gene (encoding GS type I, here referred to as GS) and the *gifA* gene (encoding IF7A) constitute an example of two overlapping, strongly regulated transcripts that are produced in antisense orientation. The *glnA* and *gifA* transcripts overlap tail-to-tail so that the 3′ end of the *gifA* transcript extends antisense over the *glnA* transcript (Brenes-Álvarez et al. 2023). Therefore, we considered the possibility that transcription of this antisense RNA could affect the accumulation and/or translation of the *glnA* mRNA, and posttranscriptionally modulate the levels of GS activity. The transcript that encodes *gifA* would constitute a dual RNA with two regions, a 5′ protein-coding region, encoding IF7A, and a 3′ untranslated region acting as an antisense to the *glnA* mRNA.

In this work, we show, by manipulating in vivo the amount of *glnA* antisense RNA, that this antisense RNA can modulate the level of *glnA* mRNA and GS activity. Furthermore, the overexpression in *trans* of an artificial antisense RNA to *glnA* resulted in the RNase III–dependent degradation of *glnA* mRNA. Taken together, these observations confirm the potential physiological relevance of overlapping transcription in the case of *glnA*, and provide further evidence supporting the use of antisense transcripts, provided either in *cis* or in *trans*, as tools to tune gene expression in filamentous, nitrogen-fixing cyanobacteria, which represent valuable chassis for biotechnological applications.

## Results

### Transcription of *gifA* produces an antisense to *glnA*

**Figure 1.**
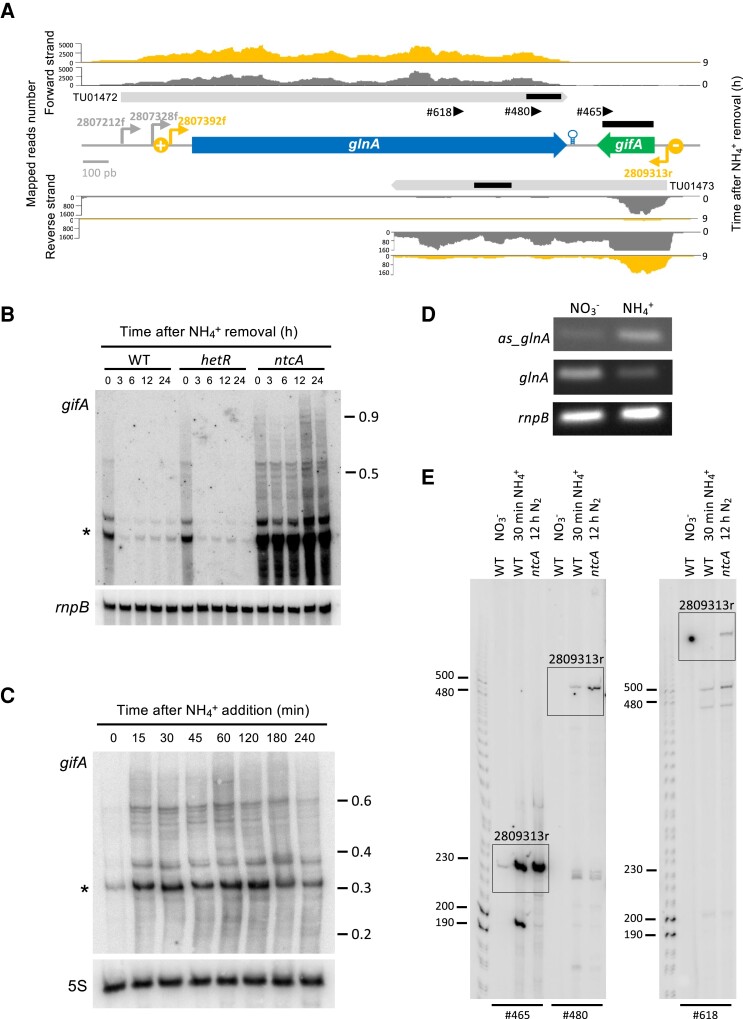
Characterization of antisense transcription overlapping *glnA*. **A)** Distribution of reads mapped in the *glnA-gifA* region obtained from cells grown in the presence of ${\mathrm{NH}}_{4}^{+}$ (0), or after 9 h (9) in the absence of combined nitrogen. Transcriptomic data were taken from Brenes-Álvarez et al. (2023). Open reading frames are represented by blue (*glnA*) or green (*gifA*) arrows. The predicted transcriptional units (TUs; Brenes-Álvarez et al. 2023) are represented by gray arrows, with their corresponding identification. The transcriptional start sites corresponding to promoters regulated by NtcA positively (*glnA*) or negatively (*gifA*) are highlighted in orange bent arrows and indicated by + or − signs, respectively. Other transcriptional start sites are indicated by gray bent arrows. The predicted transcriptional terminator of the *glnA* mRNA (Ionescu et al. 2010) is indicated by a hairpin. Genomic coordinates denote the position of the transcriptional start sites in the *Nostoc* sp. PCC 7120 chromosome. The scale indicates the number of mapped reads per nucleotide position. The region corresponding to TU01473 is also shown with the scale expanded 10-fold to better visualize coverage in the region antisense to *glnA*. The bar above *gifA* indicates the probe used in the northern blots shown in **B** and **C**. The bars inside the arrows indicate the regions of the *glnA* and *as_glnA* amplified by the PCRs shown in **D**. The triangles indicate the position of the primers used for the primer extension assays shown in **E**. **B)** Northern blot of RNA extracted from wild-type cells (WT), from an *hetR* mutant strain, or from a *ntcA* mutant strain grown in the presence of ${\mathrm{NH}}_{4}^{+}$, or after the indicated times in the absence of combined nitrogen, fractionated in a 3% urea polyacrylamide gel and hybridized with a probe for *gifA*. *rnpB* was used as a loading and transfer control. **C)** Northern blot of RNA extracted from wild-type cells grown in the absence of combined nitrogen or at the indicated times after addition of ${\mathrm{NH}}_{4}^{+}$, fractionated in a 6% urea-polyacrylamide gel and hybridized with a probe for *gifA*. 5S RNA was used as the loading and transfer control. **D)** Expression of *glnA* and *as_glnA* according to semiquantitative RT-PCR in wild-type cells growing in the presence of ${\mathrm{NO}}_{3}^{-}$ or after 2 h of ${\mathrm{NH}}_{4}^{+}$ addition. The amplified RNA segments are indicated with black bars in **A**. *rnpB* was used as a control. **E)** Primer extension assays with RNA extracted from WT cells growing in the presence of ${\mathrm{NO}}_{3}^{-}$, or 30 min after ${\mathrm{NH}}_{4}^{+}$ addition, or from an *ntcA* mutant 12 h after combined nitrogen depletion. The primers used for extension are indicated at the bottom of each gel, and their positions are indicated by black triangles in **A**. The bands corresponding to primer extension products that end at the transcriptional start of *gifA* (position 2809313r) are boxed. Size markers are indicated in kb **(B**, **C)** or in nucleotides **(E)**. The asterisks in **B** and **C** denote the major transcript hybridizing with a *gifA* probe.

In *Nostoc* sp. PCC 7120, the *glnA* and *gifA* genes are oriented tail to tail. In our previous work (Brenes-Álvarez et al. 2023), we have shown that the corresponding transcripts overlap, and in the presence of ammonium (a condition in which *gifA* is highly expressed), a substantial fraction of the *gifA* transcript extends beyond the *gifA* coding sequence, antisense to the *glnA* transcript (Fig. 1A). We have confirmed the presence of RNA antisense to *glnA* in several ways. Northern blots hybridized with a *gifA* probe showed a major band that would correspond to transcriptional termination closely after the end of the *gifA* coding sequence (band of about 300 nucleotides, marked with an asterisk in Fig. 1, B and C). However, some transcripts extend much longer (bands up to about 900 nucleotides, Fig. 1, B and C). By semiquantitative RT-PCR, we could also detect an antisense RNA for *glnA* that accumulated with the addition of ammonium to the cultures (Fig. 1D). Finally, by primer extension with primers within the *glnA* coding region (#480 and #618), we could demonstrate that, upon *gifA* transcription derepression, there are transcripts that extend from the *gifA* promoter to well within the coding sequence of *glnA* (Fig. 1E). Extended products are more readily observed in the case of the *ntcA* mutant strain, which, in the absence of repression by NtcA, shows constitutive strong expression of *gifA* irrespective of the nitrogen source (Galmozzi et al. 2010; Fig. 1, B and E). Also, note that primer extension depends on the integrity of the transcript acting as a template; therefore, the observation of bands corresponding to the *gifA* TSS (2809313r) implies the presence of RNA molecules that extend from the position of the TSS to, at least, the position of the oligonucleotide used in each extension reaction. All of these combined results indicate that a significant fraction of the *gifA* transcript extends antisense to *glnA* mRNA.

### Transcription of an antisense to *glnA* affects GS activity

**Figure 2.**
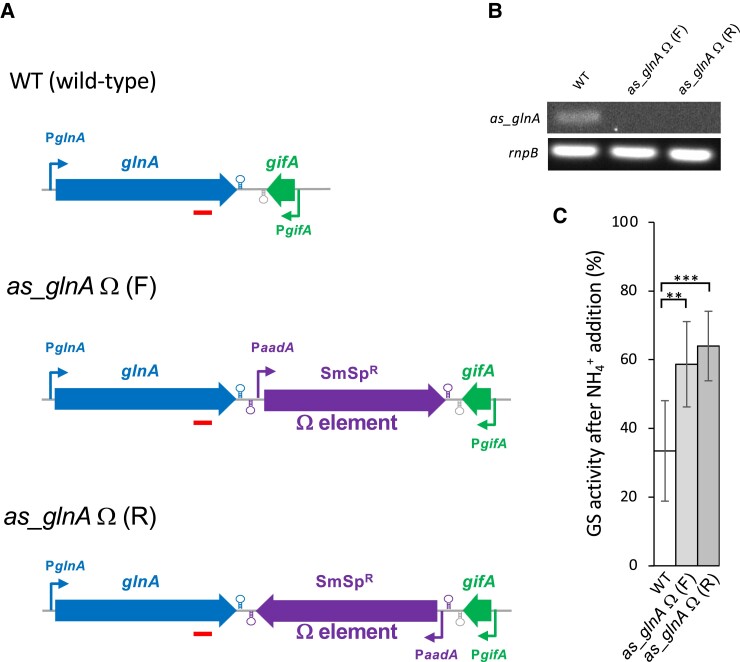
Effect of blocking antisense expression on GS regulation by ammonium. **A)** Scheme of the *glnA-gifA* region in the wild-type (WT) strain, and in strains with an antibiotic resistance cassette flanked by transcription terminators inserted between *glnA* and *gifA* in the forward orientation (*as_glnA* Ω (F)) or in the reverse orientation (*as_glnA* Ω (R)) with respect to *glnA*. Promoters are indicated by bent arrows, and transcription terminators are indicated by hairpins. The bars under the *glnA* gene indicate the region amplified in the RT-PCR shown in **B**. **B)** Expression of the *glnA* antisense RNA according to semiquantitative RT-PCR in ${\mathrm{NO}}_{3}^{-}$ grown cells of the wild type, strain *as_glnA* Ω (F), and strain *as_glnA* Ω (R) 2 h after ${\mathrm{NH}}_{4}^{+}$ addition. *rnpB* was used as a control. **C)** Reduction of GS activity upon addition of ${\mathrm{NH}}_{4}^{+}$ in wild type, strain *as_glnA* Ω (F), or strain *as_glnA* Ω (R) cells. GS activity (U/mg chlorophyll) was measured in cells growing in the presence of ${\mathrm{NO}}_{3}^{-}$ and 2 h after the addition of ${\mathrm{NH}}_{4}^{+}$. The plot represents the percentage of GS activity in the cells 2 h after ${\mathrm{NH}}_{4}^{+}$ addition compared with ${\mathrm{NO}}_{3}^{-}$ grown cells before ${\mathrm{NH}}_{4}^{+}$ addition. Average and Sd of the quantification of 7 (WT) or 8 (*as_glnA* Ω (F) and *as_glnA* Ω (R)) biological replicates. **P < 0.01; ***P < 0.001, Student's *t* test.

We hypothesized that the antisense portion of the *gifA* transcript might have an effect on the expression of *glnA* and contribute to the regulation of GS. To test this hypothesis, we prepared two strains in which the Ω element was inserted, in both orientations, between the *glnA* and *gifA* coding sequences [strains *as_glnA* Ω (F) and *as_glnA* Ω (R), Fig. 2A]. The Ω element consists of a SmSp^R^ gene (*aadA*) flanked by short inverted repeats carrying transcription and translation termination signals (Prentki and Krisch 1984). We therefore expected that in strains *as_glnA* Ω (F) and *as_glnA* Ω (R), there would be no transcription antisense to *glnA*, because the terminators flanking the antibiotic resistance gene would block read through from the *gifA* promoter. We confirmed this prediction by semiquantitative RT-PCR upon ammonium addition, which leads to derepression of the *gifA* promoter (Fig. 2B). While in the wild-type strain, the antisense to *glnA* is readily detectable, no detectable amount of antisense RNA was observed in strains *as_glnA* Ω (F) or *as_glnA* Ω (R). This result confirms that the *glnA* antisense detected by RT-PCR in Fig. 1D is a product of the read through transcription from the *gifA* promoter and is not associated with another undetected antisense transcriptional start site inside of *glnA.* Then, we analyzed the GS activity in these strains (Fig. 2C). It is well established that upon ammonium addition, there is a reduction in GS activity, which can be attributed to the inhibitory effect of IF7, the product of the *gifA* gene, whose transcription is derepressed under these conditions (Galmozzi et al. 2010). Figure 2C shows that, as expected, in the wild-type strain GS activity (normalized according to chlorophyll content as indicated in the Materials and Methods section) drops by >60% 2 h after ammonium addition compared with the GS activity measured in the cells before ammonium addition (grown in the presence of ${\mathrm{NO}}_{3}^{-}$). In contrast, in strains *as_glnA* Ω (F) and *as_glnA* Ω (R), in which the transcript that originates from the *gifA* promoter does not reach the *glnA* coding sequence, the decrease in GS activity is significantly lower, around 40%. This result strongly suggests that, at least part of the decrease in GS activity observed upon ammonium addition in the wild-type strain may be due to the presence of an antisense to the *glnA* mRNA, which is absent in strains *as_glnA* Ω (F) and *as_glnA* Ω (R).

**Figure 3.**
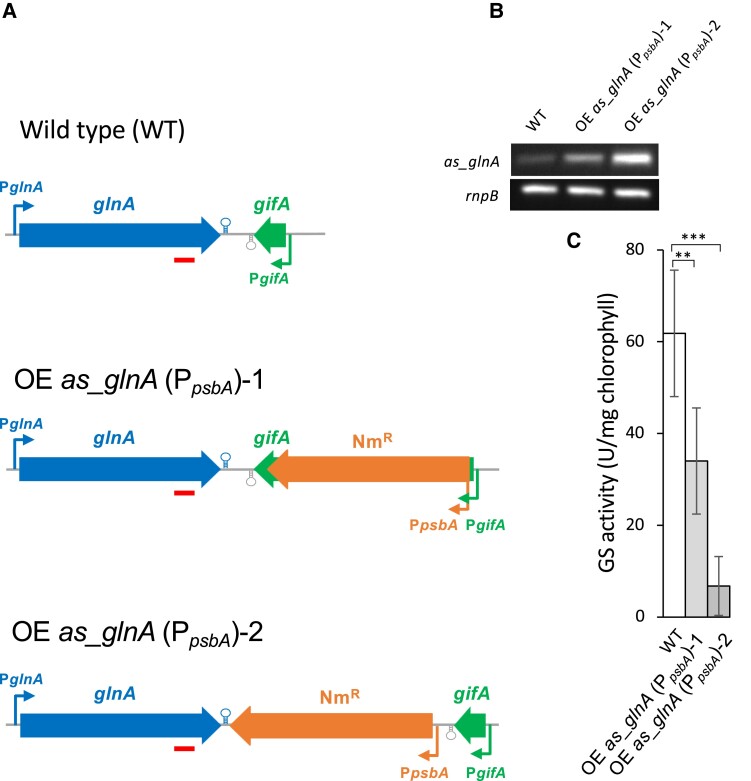
Effect of antisense overexpression on GS expression. **A)** Scheme of the *glnA-gifA* region in the wild-type (WT) strain, and in strains with an antibiotic resistance cassette lacking transcription terminators inserted within *gifA* (strain OE *as_glnA* (P*_psbA_*)-1) or between *glnA* and *gifA* (strain OE *as_glnA* (P*_psbA_*)-2). Promoters are indicated by bent arrows, and transcription terminators are indicated by hairpins. The bars under the *glnA* gene indicate the region amplified in the RT-PCR shown in **B**. **B)** Expression of the *glnA* antisense RNA according to semiquantitative RT-PCR in wild type, strain OE *as_glnA* (P*_psbA_*)-1, and strain OE *as_glnA* (P*_psbA_*)-2 cells grown in the presence of ${\mathrm{NO}}_{3}^{-}$ containing media. *rnpB* was used as a control. **C)** GS activity of wild type, strain OE *as_glnA* (P*_psbA_*)-1, and strain OE *as_glnA* (P*_psbA_*)-2 grown in the presence of ${\mathrm{NO}}_{3}^{-}$. Average and Sd of the quantification of 10 (WT), 9 (OE *as_glnA* (P*_psbA_*)-1), or 12 (OE *as_glnA* (P*_psbA_*)-2B) biological replicates. **P < 0.001; ***P < 0.0001, Student's *t* test). OE, overexpressor.

Next, we tested whether increased expression of the *glnA* antisense RNA would further reduce the activity of GS. For this purpose, we used an Nm^R^ cassette bearing the strong *psbA* promoter of *Amaranthus hybridus* and lacking a transcriptional terminator (Elhai and Wolk 1988a). We inserted such cassette either interrupting the *gifA* coding sequence (strain OE *as_glnA* (P*_psbA_*)-1) or in the intergenic region between the *glnA* and the *gifA* coding sequences (strain OE *as_glnA* (P*_psbA_*)-2). In both cases, the Nm^R^ gene in the cassette is transcribed from the strong *psbA* promoter in the opposite direction to *glnA* (Fig. 3A). Although in strain OE *as_glnA* (P*_psbA_*)-1, transcription would mostly be terminated at the transcriptional terminator observed downstream of *gifA* (indicated by a gray hairpin in Fig. 3A), in strain OE *as_glnA* (P*_psbA_*)-2, the cassette was inserted between the region where strong transcription termination was observed for the *gifA* mRNA and the transcriptional terminator predicted for the *glnA* mRNA (indicated by a blue hairpin in Fig. 3A). Therefore, we expected stronger antisense transcription in strain OE *as_glnA* (P*_psbA_*)-2 than in strain OE *as_glnA* (P*_psbA_*)-1. We analyzed the amount of *glnA* antisense RNA by semiquantitative RT-PCR in cells grown in ${\mathrm{NO}}_{3}^{-}$-containing medium (Fig. 3B), a condition in which *as_glnA* produced from the native *gifA* promoter is hardly detected (see Fig. 1E). Both strains had higher amounts of antisense RNA than the wild type, with a much stronger signal in strain OE *as_glnA* (P*_psbA_*)-2, as expected. We then measured the GS activity in these cells. Consistent with the results shown in Fig. 3B, lower levels of GS activity were measured in strains OE *as_glnA* (P*_psbA_*)-2 and OE *as_glnA* (P*_psbA_*)-1 than in wild type (Fig. 3C), indicating that an antisense RNA transcribed in *cis* opposite to *glnA* can have an inhibitory effect on GS expression. The inhibition was stronger in strain OE *as_glnA* (P*_psbA_*)-2, consistent with a higher amount of antisense RNA, than in strain OE *as_glnA* (P*_psbA_*)-1.

### GS activity can be modulated by an antisense RNA transcribed in *trans*

**Figure 4.**
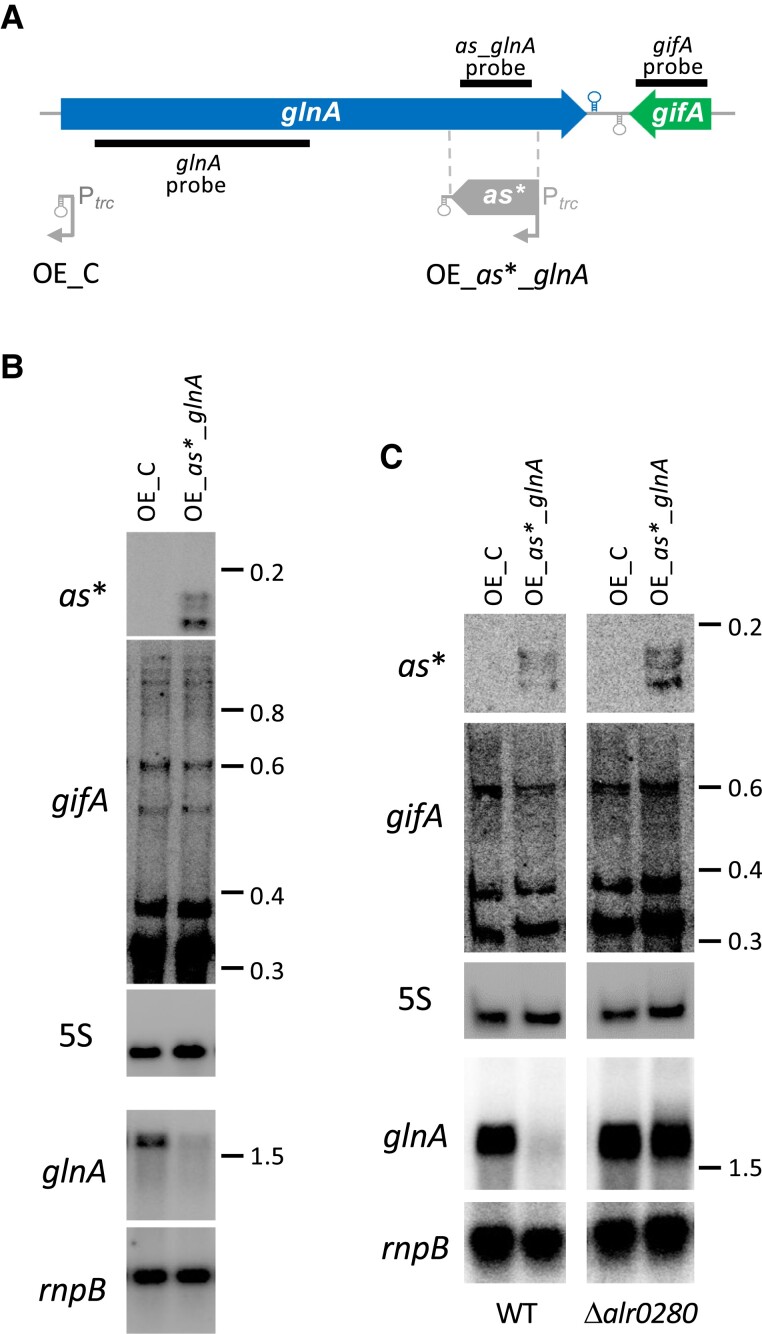
Overexpression in *trans* of an antisense RNA to *glnA*. **A)** Diagram of the *glnA-gifA* region. The bars indicate the positions of the probes used in the northern blots shown in **B** and **C**. The position of the antisense RNA (OE_as*_*glnA*) overexpressed from the *trc* promoter in *trans* is indicated. As a control, a strain overexpressing only the terminator sequence was used (OE_C). **B)** Northern blot with RNA extracted from OE_C or OE_as*_*glnA* cells 2.5 h after ${\mathrm{NH}}_{4}^{+}$ addition. **C)** Northern blot with RNA extracted from OE_C or OE_as*_*glnA* cells from wild type (left, WT) or RNase III mutant strain Δ*alr0280* (right) grown in the presence of ${\mathrm{NO}}_{3}^{-}$. In **B** and **C**, the RNA was subjected to electrophoresis in 4% **(B)** or 6% **(C)** acrylamide gels (top 3 panels) and hybridized sequentially with an as*_*glnA* probe, a *gifA* probe, and a 5S probe, or in 1% agarose gel (bottom 2 panels), and hybridized sequentially with a *glnA* probe and a *rnpB* probe. 5S and *rnpB* were used as load and transfer controls. OE, overexpressor.

Our results so far indicate that the transcription antisense to *glnA* either from the native (nitrogen-regulated) *gifA* promoter (Fig. 2) or from a constitutive *psbA* promoter introduced downstream of the *glnA* gene (Fig. 3) results in a reduction of GS expression. Regulation by an antisense transcript supplied in *trans* has been used for artificial inhibition of gene expression, including potentially interesting biotechnological targets. To analyze the potential of antisense inhibition in the reduction of GS expression in *Nostoc*, we introduced by conjugation and single recombination a portion of the sequence corresponding to the *glnA* antisense (indicated as as*_*glnA*) under the control of the strong, constitutive *trc* promoter (strain OE_as*_*glnA*), followed by the *T1* transcriptional terminator (Fig. 4A). The plasmid bearing this construct would integrate at a neutral site in plasmid alpha (Olmedo-Verd et al. 2006), leading to transcription of as*_*glnA* in *trans* with respect to *glnA*. As a control, the previously described strain bearing pMBA51 (Olmedo-Verd et al. 2019) was used (strain OE_C). In this strain, only the *T1* transcription termination sequence is transcribed. We confirmed by northern blot that strain OE_as*_*glnA* accumulates antisense RNA, and this accumulation is associated with a strong reduction in *glnA* transcript accumulation regardless of the presence of ${\mathrm{NH}}_{4}^{+}$ and *gifA* transcription (Fig. 4B). Furthermore, we prepared strains bearing these constructs also in an RNase III mutant background (Olmedo-Verd et al. 2019). Overexpression of the antisense RNA in a strain lacking RNase III did not result in a reduction in the amount of *glnA* transcript (Fig. 4C). Therefore, it can be concluded that RNase III is responsible for the degradation of the duplex generated between *glnA* mRNA and its antisense produced in *trans*.

## Discussion

Several hints suggest that the organization of genes on the bacterial chromosome is not random, but could rather have regulatory implications. The most obvious example is the concept of operon, since placing two or more structural genes in a single transcriptional unit facilitates the regulation of these neighboring genes by a single regulatory protein (Maas and McFall 1964). Regulation of several genes by a certain transcription factor may also be facilitated by the proximity, in a 3D nucleoid organization, of the gene encoding the transcription factor and the regulated genes (Dorman 2013). The spatial distribution of genes along the chromosome may also have an impact on their expression when their transcripts overlap. Transcriptomic analysis of *Listeria monocytogenes* led to the definition of the excludon concept, in which the expression of an mRNA has regulatory consequences on the expression of an overlapping, divergently oriented gene (Wurtzel et al. 2012). The paradigm of excludon transcriptional organization extends to other bacteria, including cyanobacteria, and provides a simple way to achieve coordinated expression of adjacent genes often encoding proteins of related or potentially antagonistic functions (Sesto et al. 2013; Georg and Hess 2018).

In this work, we show that transcription antisense to *glnA* (originating from the *gifA* promoter upon derepression of *gifA* expression) has an impact on GS expression in *Nostoc* sp. PCC 7120, in which *glnA* and *gifA* are oriented tail to tail. Although most *gifA* transcripts terminate shortly after the IF7 coding sequence, there is substantial read through that results in the accumulation of antisense transcripts to *glnA*. Blocking antisense transcription without affecting the *gifA* coding sequence results in a reduced decrease in GS activity after the addition of ${\mathrm{NH}}_{4}^{+}$ (Fig. 2). Furthermore, increased antisense transcription from a strong promoter results in reduced GS activity (Fig. 3). Given the observation that about half of the decrease in GS activity after ammonium addition is lost in the strains in which antisense regulation is abolished (Fig. 2C), we can conclude that the impact of antisense regulation on GS activity in *Nostoc* is not minor.

The antisense regulation described here could be exerted through the coupled degradation of RNA duplexes by RNAse III. In fact, we have shown that, when *as_glnA* is provided in trans, *glnA* mRNA is degraded in an RNase III–dependent manner (Fig. 4C). Furthermore, a recent report has shown that transcriptional interference due to a tail-to-tail disposition could be prevalent in prokaryotic transcriptomes (Wanney et al. 2023). Given the tail-to-tail disposition of the 2 mRNAs involved (*glnA* and *gifA*), we cannot exclude that this transcriptional interference phenomenon may participate in the regulation operated by the native antisense RNA in the case of *glnA*.

Supplementary Table S1 shows the distribution of *gifA* and *gifB* (DUF4278) genes encoding IFs in a selection of cyanobacterial genomes (Shih et al. 2013) included in the RefSeq database. The presence of several genes encoding IF homologs, as in the case of the unicellular strain *Synechocystis* sp. PCC 6803, seems to be more extended than the presence of a single *gifA* gene, as in the case of the filamentous strain *Nostoc* sp. PCC 7120. In the best studied case, the *Synechocystis* sp. PCC 6803, each of its two IFs (IF7 and IF17) has been shown to contribute to GS inactivation in vivo, so that a maximal level of inactivation by ammonium addition is observed when both proteins are present (García-Domínguez et al. 1999). In this context, we speculate that in strains with only one IF, such as those in the Nostocales group (Supplementary Table S1), an additional posttranscriptional regulatory mechanism could have evolved in which RNA–RNA interactions derived from the antisense disposition of the *glnA* and the *gifA* genes can additionally contribute to regulating the accumulation of the *glnA* transcript at the posttranscriptional level. The tail-to-tail disposition of *glnA* and *gifA* that seems to be exclusive of strains in the Nostocales group strongly correlates with the absence of a second gene encoding an IF (*gifB*; see Supplementary Table S1 and Fig. S1). The antisense regulatory mechanism involving the *glnA* and *gifA* mRNAs that we describe here could be widespread among Nostocales, given the tail-to-tail disposition of these two genes in these strains. This genomic arrangement may have additional regulatory implications. Translation of some mRNAs occurs close to the DNA template in *Escherichia coli* and *Caulobacter crescentus* (Montero Llopis et al. 2010). If this were the case for the *glnA* and *gifA* mRNAs, given that Nostocales cells are larger than unicellular cyanobacterial cells and they contain some of the largest genomes in the prokaryotic world (Hess 2011), the translation of GS and IF right next to each other could facilitate a fast coupled regulation by protein–protein interaction.

**Figure 5.**
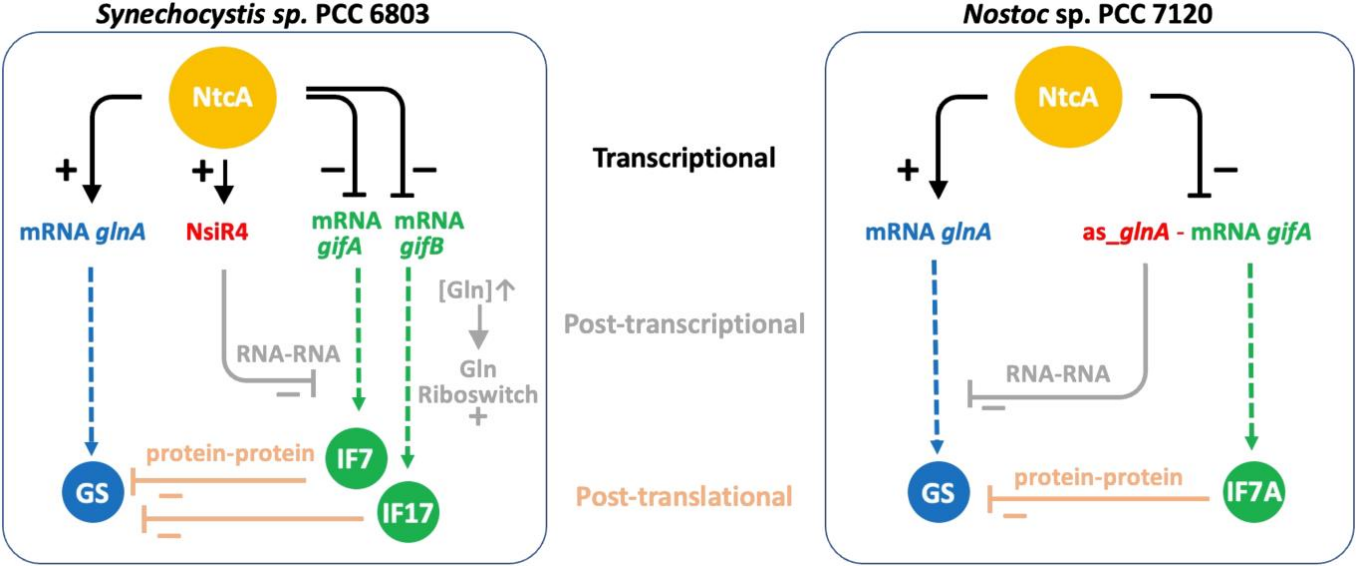
Comparative model of the regulation of GS in *Nostoc* and *Synechocystis*. Three levels of regulation (transcriptional, posttranscriptional, and posttranslational) are indicated. The inverse regulation of GS and IFs at the transcriptional level by NtcA is indicated with black arrows and T bars. Arrows indicate a positive effect, and T bars indicate a negative effect. Posttranscriptional regulation by RNA–RNA interactions is indicated in gray. In *Synechocystis*, the NtcA induced small noncoding RNA NsiR4 inhibits *gifA* mRNA translation (Klähn et al. 2015), while a glutamine riboswitch activates IF17 translation in response to glutamine (Klähn et al. 2018). In *Nostoc*, the *glnA* antisense region of the *gifA* mRNA reduces the expression of *glnA* (this work). Dashed lines represent the translation of GS or the IF factors. Finally, posttranslational regulation is operated by IFs (IF7A in *Nostoc*; IF7 and IF17 in *Synechocystis*) that inactivate GS by direct protein–protein interaction (García-Domínguez et al. 1999; Galmozzi et al. 2010).

Figure 5 shows a comparison of the different levels of transcriptional, posttranscriptional, and posttranslational regulation described for GS in *Synechocystis* sp. PCC 6803, bearing the two IFs IF7 and IF17, and *Nostoc* sp. PCC 7120, which encodes only one IF7 homolog, IF7A. Recent findings in cyanobacteria have demonstrated the existence of versatile riboregulatory mechanisms that modulate the C/N balance (Muro-Pastor and Hess 2020). The negative effect of the NtcA-regulated small RNA NsiR4 on the accumulation of IF7 constitutes an example of posttranscriptional regulation operated by a noncoding RNA (Klähn et al. 2015). Similarly, in the case of *Nostoc* sp. PCC 7120, the regulation described here exerted by the *gifA* mRNA as an antisense of the *glnA* mRNA also operates by RNA–RNA interaction.

The recent definition of the transcriptome of *Nostoc* sp. PCC 7120 (Brenes-Álvarez et al. 2023) reveals that many transcriptional units produced from oppositely oriented adjacent genes extend into each other, therefore producing antisense transcripts that occasionally cover a significant portion of the corresponding coding sequences. The possible regulatory consequences of such overlap must be determined in each case, but in the case of strongly regulated transcripts, one can envision that the regulated expression of a certain gene can indirectly affect the expression of its overlapping partner. As described here, this seems to be the case for the oppositely oriented transcriptional units containing the *glnA* and the *gifA* genes, both of which are regulated in opposite ways by the availability of nitrogen.

The observation that a reduction in GS activity can be achieved by increasing, either in *cis* (Fig. 3) or in *trans* (Fig. 4), the amount of *glnA* antisense above the level produced in the wild-type strain demonstrates the possibility of using antisense transcription as a tool for pathway engineering in *Nostoc*. For this purpose, trans-encoded antisense RNAs could be transcribed from DNA stably integrated in a neutral site of the genome, as described here in the case of plasmid alpha, or from high copy replicative plasmids producing higher levels of antisense RNA from constitutive or regulated promoters. In this context, cell-specific promoters that operate either in vegetative cells or in heterocysts could be used for pathway engineering in specific cell types. Cyanobacteria have great potential as solar-driven cell factories and several approaches to modulate expression of essential metabolic enzymes have been tested in order to modify their performance as biotechnological chassis, including repression of endogenous genes by a small noncoding RNA (Higo et al. 2016) or by the use of CRISPR interference (Higo et al. 2018; Shabestary et al. 2018). As described here for the case of GS, optimizing native RNA-mediated regulatory mechanisms could help achieve metabolic reprogramming and redirection of fluxes to desired pathways.

## Materials and methods

### Strains and growth conditions

The strains used in this work are described in Supplementary Table S2. Cultures used in the northern blots and primer extension assays included in Fig. 1 were bubbled with an air/CO_2_ mixture (1% v/v) and grown photoautotrophically at 30°C in BG11 medium (Rippka et al. 1979) containing ferric citrate instead of ammonium ferric citrate and 10 mM NaHCO_3_. About 17.6 mM NaNO_3_ or 6 mM NH_4_Cl plus 12 mM *N*-tris (hydroxymethyl) methyl-2-aminoethanesulfonic acid-NaOH buffer (pH 7.5) was used as nitrogen sources. Cultures used in the rest of the experiments were grown in flasks using the same media without NaHCO_3_ or air/CO_2_ bubbling. The addition of 10 mM NH_4_Cl plus 20 mM *N*-tris (hydroxymethyl) methyl-2-aminoethanesulfonic acid-NaOH buffer (pH 7.5) was used to derepress the expression of the *gifA* mRNA. Nitrogen deficiency was induced by filtering, washing, and resuspending cells in nitrogen-free BG11 medium.

The *Nostoc* strains containing SmSp^R^ genes were grown in the presence of streptomycin (Sm) and spectinomycin (Sp), 1.5 *µ*g/mL each (liquid medium) or 3 *µ*g/mL each (solid medium), while those containing Nm^R^ genes were grown in the presence of 5 *µ*g/mL neomycin (liquid medium) or 25 *µ*g/mL neomycin (solid medium). The medium was solidified by adding of 10 g/L of Bacto Agar (BD). *E. coli* strains were grown in LB medium, supplemented with appropriate antibiotics (Sambrook and Russell 2001).

### Construction of *Nostoc* sp. PCC 7120 derivative strains

The plasmids and oligonucleotides used in this work are described in Supplementary Tables S3 and S4, respectively. All PCR fragments were amplified with high fidelity iProof DNA polymerase. The sequences of all PCR-amplified fragments were verified entirely by sequencing.

Several plasmids were constructed to interrupt the *glnA*-*gifA* region in the *Nostoc* sp. PCC 7120 genome using antibiotic resistance cassettes. The Nm^R^ cassette C.K3 bearing the promoter of the *psbA* gene of *A. hybridus* (Elhai and Wolk 1988a) or SmSp^R^ Ω fragment (Prentki and Krisch 1984; Elhai and Wolk 1988a) were used for this purpose. First, a BamHI site was created between the coding regions of *glnA* and *gifA*, and a mutation was introduced in the start codon of *gifA* by overlapping PCR amplification with oligonucleotides 1019 + 1020, 1021 + 1024, and 1022 + 1023 using genomic DNA as a template. The change in the start codon is necessary to prevent the expression of IF7, which seems to be toxic in *E. coli* (I.Á.-E. and A.M.M-P., unpublished data), during intermediate cloning steps. These three segments were used as templates and combined in a final segment amplified with oligonucleotides 1019 + 1022 that was cloned in the pSparkII vector, rendering pIAE86. The BamHI fragments containing the Nm^R^ cassette C.K3 (from pMBA85) or SmSp^R^ Ω cassette C.S3 (from pRL463) were inserted in BamHI-digested pIAE86 at the BamHI site created between *glnA* and *gifA*, rendering pIAE88 (Nm^R^ gene in the C.K3 cassette in the same orientation of *gifA*) or pIAE89a/b (SmSp^R^ gene in the C.S3 cassette in both orientations), respectively. The XhoI fragments containing the interrupted *glnA*-*gifA* regions from pIAE88 or pIAE89a/b were cloned into pRL277 or pRL278 digested by XhoI, respectively, rendering pIAE90 and pIAE91a/b.

The Nm^R^ cassette C.K3 was introduced within the *gifA* coding region as follows. Two segments were amplified by PCR using genomic DNA as a template and oligonucleotides 1025 + 1026 and 1027 + 1028. Both fragments, ligated at their BamHI site, were cloned in the pSparkII vector rendering pIAE79. In the resulting cloned region, 96 nt of the *gifA* coding sequence were deleted and replaced by a BamHI site. The Nm^R^ cassette C.K3, extracted as a BamHI fragment from pMBA85, was introduced into pIAE79 digested with BamHI, rendering pIAE82 (Nm^R^ gene in the C.K3 cassette in the same orientation of *gifA*). The XhoI fragment containing the *glnA*-*gifA* region interrupted with the C.K3 casette from pIAE82 was cloned into pRL277 digested by XhoI rendering pIAE85.

pIAE85, pIAE90, pIAE91a, and pIAE91b were introduced in *Nostoc* by conjugation (Elhai and Wolk 1988b) and double recombinants were selected, rendering strains OE *as_glnA* (P*_psbA_*)-1, OE *as_glnA* (P*_psbA_*)-2, *as_glnA* Ω (F), and *as_glnA* Ω (R), respectively. Genomic DNA was extracted from clones exhibiting sucrose resistance and resistance to neomycin (in the case of pIAE85 and pIAE90) or spectinomycin and streptomycin (in the case of pIAE91a/b) (double recombinants). The segregation of the introduced mutations was analyzed by PCR using appropriate oligonucleotides. Clones in which the introduced constructs had replaced the wild-type *glnA*-*gifA* region in the chromosome were sequenced to verify the insertion of the cassettes and the presence of the original start codon of *gifA*.

We have used pMBA37 (Olmedo-Verd et al. 2019) as a backbone for overexpressing as*_*glnA* from the *trc* promoter. The sequence encoding a segment of *as*_*glnA* was amplified using genomic DNA as a template and oligonucleotides 613 + 616. The products were digested with NsiI and XhoI and cloned between the NsiI and XhoI sites in pMBA37 (between the *trc* promoter and the *T1* terminator of the *E. coli rrnB* gene), rendering pSAM336. pMBA51, a plasmid that overexpresses a control RNA corresponding only to the *T1* terminator under the *trc* promoter (Olmedo-Verd et al. 2019), and pSAM336 were introduced in *Nostoc* wild-type and Δ*alr0280* (Olmedo-Verd et al. 2019) strains by conjugation (Elhai and Wolk 1988b) generating strains OE_C and OE_as*_*glnA*, respectively, that contain pMBA51 or pSAM336 inserted in the alpha megaplasmid from *Nostoc* wild-type or RNase III mutant backgrounds.

### RNA isolation and analysis

Total RNA was isolated using hot phenol as described (Mohamed and Jansson 1989) with modifications (Brenes-Álvarez et al. 2016). When appropriate, samples were treated with RNase-free DNase, according to the instructions of the TURBO DNA-free kit. Northern blot hybridization was carried out according to standard procedures after RNA separation in denaturing formaldehyde agarose gels or in urea-polyacrylamide gels (Muro-Pastor et al. 1999; Steglich et al. 2008), using ^32^P-labeled probes. The ^32^P-labeled strand–specific probes were prepared with Taq DNA polymerase using a PCR fragment (oligonucleotides indicated in Supplementary Table S4) as template in a reaction with α-^32^P-dCTP and a single oligonucleotide as primer (corresponding to the complementary strand of the RNA to be detected). Hybridization with *rnpB* (Vioque 1992) or 5S rRNA probes was used as a loading and transfer control. Primer extension analysis was performed as previously described (Muro-Pastor et al. 1999) using the oligonucleotides indicated in Supplementary Table S4 labeled with γ-^32^P-ATP. Images of radioactive gels and membranes were obtained and analyzed using a Cyclone Storage Phosphor System and OptiQuant image analysis software (Packard). For semiquantitative RT-PCR, synthesis of first strand cDNA was performed with Superscript III following the manufacturer's instructions, with 1 *µ*g of total RNA as template and using the primers 1223 for *glnA* mRNA, 1222 for *as_glnA* RNA, and 1209 for *rnpB* (Supplementary Table S4) in a total volume of 20 *µ*L. After dilution to 30 *µ*L and desalting, 1 *µ*L of the reverse transcription product was used as a template in the PCRs. PCR (25 amplification cycles) was performed using primer pairs 1124 + 1125 for *glnA* mRNA, 480 + 1207 for *as_glnA* RNA, and 1226 + 1227 for *rnpB* (Supplementary Table S4).

### Glutamine synthetase assay

GS activity was determined in situ using the Mn^2+^-dependent γ-glutamyltransferase assay in cells permeabilized with mixed alkyltrimethylammonium bromide (MTA; Mérida et al. 1991). One unit of GS activity corresponds to the amount of enzyme that catalyzes the synthesis of 1 *μ*mol min^−1^ of γ-glutamylhydroxamate. GS activity was normalized between samples according to the chlorophyll content of the cells used.

### Computational and statistical methods

The synteny of genes around *glnA* was analyzed with webFlaGs (Saha et al. 2021). The genomes selected were those used in Shih et al. (2013) that are completely assembled.

Homologs of *glnA* and *gifA* were identified by PSI-BLAST (Altschul et al. 1997). Homologs of *gifB* were identified in the Pfam database (Mistry et al. 2021) as homologs to DUF4278.

The Student's *t* test was used to determine statistical significance. The number of replicates can be found in the corresponding figure legends.

## Accession numbers

Sequence data used in this article can be found in the GenBank/EMBL data libraries under accession number NC_003272.1.

## Supplementary Material

kiae263_Supplementary_Data

## Data Availability

The data underlying this article are available in the article and in its online supplementary material.

## Supplementary data

The following materials are available in the online version of this article.

**Supplementary Figure S1.** Comparison of the genomic region around *glnA* in Cyanobacteria.

**Supplementary Table S1.** The presence of *gifA*, *gifB* (DUF4278), and potential antisense RNA of *glnA* in different Cyanobacteria.

**Supplementary Table S2.** Strains.

**Supplementary Table S3.** Plasmids.

**Supplementary Table S4.** Oligonucleotides.
