# Supplementary material for: Antisense RNA regulates glutamine synthetase in a heterocyst-forming cyanobacterium
Source: Plant Physiol. 2024 May 6;195(4):2911–20. doi: 10.1093/plphys/kiae263 (PMC11288750; doi:10.1093/plphys/kiae263)
Supplement: kiae263_Supplementary_Data [file kiae263_supplementary_data.zip › AlvarezEscribano_SI_280424.pdf]

Supplementary Materials for:

Antisense RNA regulates glutamine synthetase in a heterocyst-forming cyanobacterium

Isidro Álvarez-Escribano, Belén Suárez-Murillo, Manuel Brenes-Álvarez, Agustín Vioque, and Alicia M. Muro-Pastor*

This PDF includes:

- Supplementary Figure S1
- Supplementary Tables S1 to S4
- References for supplementary material citations

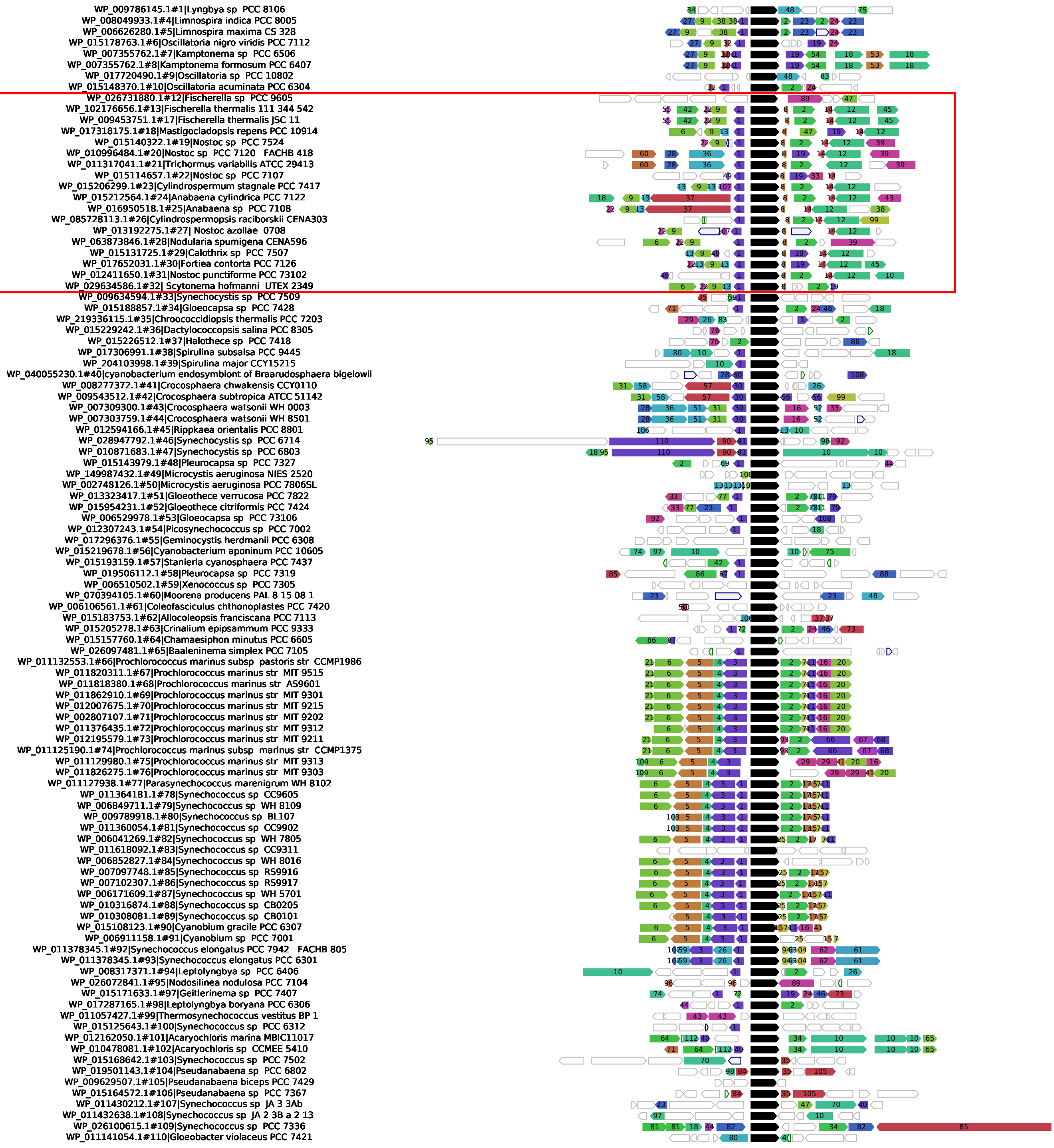

Supplementary Figure S1. Comparison of the genomic region around *glnA* in Cyanobacteria. The gene synteny around *glnA* (black arrows) was analyzed as described in Materials and Methods. The red frame highlights Nostocales strains. The brown arrowheads immediately downstream of *glnA* (and labelled 8) represent *gifA* homologs.

Supplementary Table S1. Presence of *gifA*, *gifB* (DUF4278), and potential antisense RNA of *glnA* in different Cyanobacteria

**Organism**	***gifA***[a]	***gifB***[b]	**antisense**[c]
Lyngbya sp PCC 8106	2	2	-
Arthrospira platensis str Paraca	3	2	-
Arthrospira platensis NIES 39	3	2	-
Limnospira indica PCC 8005	2	2	-
Limnospira maxima CS 328	3	2	-
Oscillatoria nigro viridis PCC 7112	1	1	-
Kamptonema sp PCC 6506	1	3	-
Kamptonema formosum PCC 6407	1	3	-
Oscillatoria sp PCC 10802	2	2	-
Oscillatoria acuminata PCC 6304	1	3	-
Fischerella muscicola SAG 1427 1 PCC 73103	1	2	+
Fischerella sp PCC 9605	1	3	-
Fischerella thermalis 111/344/542	1	2	+
Fischerella sp NIES-4106	1	3	+
Fischerella sp PCC 9431	1	2	-
Fischerella sp PCC 9339	1	3	+
Fischerella thermalis JSC-11	1	2	+
Mastigocladopsis repens PCC 10914	1	2	+
Nostoc sp. PCC 7524	2	0	+
Nostoc sp. PCC 7120	1	0	+
Trichormus variabilis ATCC 29413	1	0	+
Nostoc sp. PCC 7107	1	0	+
Cylindrospermum stagnale PCC 7417	1	0	+
Anabaena cylindrica PCC 7122	1	0	+
Anabaena sp PCC 7108	1	0	+
Cylindrospermopsis raciborskii CENA303	1	0	+
Nostoc azollae 0708	1	0	+
Nodularia spumigena	1	0	+
Calothrix sp PCC 7507	1	0	+
Fortiea contorta PCC 7126	1	0	+
Nostoc punctiforme PCC 73102	1	0	+
Scytonema hofmanni UTEX 2349	1	0	+
Synechocystis sp. PCC 7509	1	0	-
Gloeocapsa sp. PCC 7428	1	1	-
Chroococcidiopsis thermalis PCC 7203	1	1	-
Dactylococcopsis salina PCC 8305	1	2	-
Halothece sp. PCC 7418	1	2	-
Spirulina subsalsa PCC 9445	1	2	-
Spirulina major CCY15215	1	3	-
Crocosphaera chwakensis CCY0110	1	2	-
Crocosphaera subtropica ATCC 51142	1	3	-
Crocosphaera watsonii WH 0003	1	1	-
Rippkaea orientalis PCC 8801	1	2	-
Synechocystis sp. PCC 6714	1	1	-
Synechocystis sp. PCC 6803	1	1	-
Pleurocapsa sp. PCC 7327	2	3	-
Microcystis aeruginosa NIES-2520	1	2	-
Microcystis aeruginosa PCC 7806	1	3	-
Gloeothece verrucosa PCC 7822	1	2	-

**Organism**	***gifA***[a]	***gifB***[b]	**antisense**[c]
Gloeothece citriformis PCC 7424	1	3	-
Gloeocapsa sp. PCC 73106	1	3	-
Synechococcus sp. PCC 7002	1	1	-
Geminocystis herdmanii PCC 6308	2	2	-
Cyanobacterium aponimum PCC 10605	2	1	-
Stanieria cyanosphaera PCC 7437	2	3	-
Pleurocapsa sp. PCC 7319	2	2	-
Xenococcus sp. PCC 7305	2	3	-
Moorena producens PAL-8-15-08-1	1	4	-
Coleofasciculus chthonoplastes PCC 7420	2	4	-
Allocoleopsis franciscana PCC 7113	1	3	-
Crinalium epipsammum PCC 9333	1	1	-
Baaleninema simplex PCC 7105	1	6	-
Synechococcus elongatus PCC 7942	1	1	-
Synechococcus elongatus PCC 6301	1	1	-
Leptolyngbya sp. PCC 6406	1	4	-
Geitlerinema sp. PCC 7407	1	2	-
Leptolyngbya boryana PCC 6306	1	1	-
Pseudanabaena sp. PCC 6802	1	1	-
Synechococcus sp. PCC 7336	2	2	-

The table contains the cyanobacteria studied in (Shih et al., 2013) with completely assembled genomes that contain at least one copy of the *gifA* gene. The heterocyst forming Nostocales are highlighted with grey shading.

[a]Number of *gifA* genes present.

[b]Number of *gifB* genes present.

[c]*gifA* oriented tail to tail (potential antisense) with *glnA* (+) or unlinked to *glnA* (-).

Supplementary Table S2. Strains

Strain	Description	Reference
Escherichia coli		
DH5α	Used for routine transformation	(Hanahan, 1983)
***Nostoc* sp.**		
PCC 7120	Wild type	Pasteur Culture Collection
216	Non-functional HetR. S179N mutation.	(Buikema and Haselkorn, 1991)
CSE2	*ntcA null* mutant (*ntcA*::C.S3)	(Frías et al., 1994)
Δ*alr0280*	RNase III deletion mutant	(Olmedo-Verd et al., 2019)
OE_C	pMBA51 inserted in plasmid alpha. *T1* terminator of *E. coli rrnB* gene expressed constitutively from the *trc* promoter.	(Olmedo-Verd et al., 2019)
as_glnA Ω (F)	$SmSp^R$ cassette C.S3, bearing transcriptional terminators, inserted between the *glnA* and *gifA* genes with *glnA* orientation (generated by conjugation and double recombination of plasmid pIAE91a).	This work
as_glnA Ω (R)	$SmSp^R$ cassette C.S3, bearing transcriptional terminators, inserted between the *glnA* and *gifA* genes with *gifA* orientation (generated by conjugation and double recombination of plasmid pIAE91b).	This work
OE *as_glnA* (P_{psbA})-1	*gifA* interrupted by Nm^R cassette C.K3 bearing the *psbA* promoter (generated by conjugation and double recombination of plasmid pIAE85).	This work
OE *as_glnA* (P_{psbA})-2	Nm^R cassette C.K3, bearing the *psbA* promoter, inserted between the *glnA* and *gifA* genes (generated by conjugation and double recombination of plasmid pIAE90).	This work
OE_*as*_glnA*	pSAM336 inserted in plasmid alpha (generated by conjugation and single recombination). as*_*glnA* transcribed from the *trc* promoter.	This work

Supplementary Table S3. Plasmids

Name	Description	Reference
pMBL-T	Cloning vector for PCR products	Promega
pSparkII	Cloning vector for PCR products	Canvax Biotech
pMBA37	Plasmid for expression of transcripts from the *trc* promoter.	(Olmedo-Verd et al., 2019)
pMBA51	Control plasmid, for expression of a 56 nt transcript derived from the *T1* terminator of *E. coli rrnB* gene from the *trc* promoter.	(Olmedo-Verd et al., 2019)
pRL277	Vector used for *sacB*-based positive selection of double recombinants in *Nostoc* sp. PCC 7120.	(Black et al., 1993)
pRL278	Vector used for *sacB*-based positive selection of double recombinants in *Nostoc* sp. PCC 7120.	(Black et al., 1993)
pRL463	Source of BamHI-ended $SmSp^R$ cassette.	(Frías et al., 2000)
pRL500	Positive selection vector with a symmetrical polylinker.	(Elhai and Wolk, 1988)
pIAE79	Two PCR fragments (oligonucleotides 1025 + 1026 and 1027 + 1028) digested with BamHI, ligated and cloned into pSparKII.	This work
pIAE82	BamHI-ended Nm^R cassette CK3 inserted into the BamHI site in pIAE79.	This work
pIAE85	XhoI fragment from pIAE82 cloned into XhoI-digested pRL277	This work
pIAE86	*glnA*-*gifA* region amplified by overlapping PCR with oligonucleotides 1019 + 1020, 1021 + 1024, 1022 + 1023 and finally 1019 + 1022, cloned in pSparkII	This work
pIAE88	BamHI-ended Nm^R cassette inserted into the BamHI site of pIAE86, in the same orientation of *gifA*.	This work
pIAE89a/b	BamHI-ended $SmSp^R$ cassette inserted into the BamHI site of pIAE86, in both orientations.	This work
pIAE90	XhoI digested fragment from pIAE88 cloned in XhoI-digested pRL277.	This work
pIAE91a/b	XhoI digested fragment from pIAE89a/b cloned in XhoI-digested pRL278.	This work
pMBA85	Source of BamHI-ended Nm^R cassette. Contains C. K3 cloned as a SalI-XhoI fragment from pRL278 into SalI-digested pRL500, amplified with oligonucleotide 500-1 (corresponding to the symmetrical region in pRL500) and finally cloned in pMBL-T.	This work
pSAM336	Expression of an *as*_glnA* segment from the *trc* promoter.	This work

Supplementary Table S4. Oligonucleotides

Name	Sequence (5´-3´)	Used for
**gl-7120-8**	AAGTTCATCGATACAGTAGG	Probe for *glnA*
**gl-7120-9**	CACCAGTAGCAACTTCGTGG	
**500-1**	ATAGGCGTATCACGAGGC	PCR of C.K3
**465**	AGCTCATCGAAGCATTACTAC	Probe for *gifA* and primer extension
**466**	TCTCGTGCCATTATGATGC	Probe for *gifA*
**480**	CGCTTTCTTAACAGATACAGG	Primer extension and RT-PCR assays for as_*glnA*
**613**	CGTCGTAATGCATAGAGAACTCATAAGG	Probe for *as*_glnA* and construction of pSAM336
**616**	GTTTTCTCGAGCACGCTTTCTTAACAG	Construction of pSAM336
**618**	GCACCAGTTAACTTGGCTTAC	Probe for *as*_glnA* and primer extension
**1019**	GTTTTCTCGAGCAGCACTGTTGGCAATC	Construction of pIAE86
**1020**	AGTTACTTGGATCCCCTCTAGTATACTTATAC	
**1021**	CTAGAGGGGATCCAAGTAACTTCAACAATGAG	
**1022**	GTTTTCTCGAGGCTGAGAAAATAGTAAGTGC	
**1023**	CTTGAATAGA**GGG**ACAGTCCTCCTAGTG	
**1024**	CACTAGGAGGACTGT**CCC**TCTATTCAAG	
**1025**	CCAAGCGTCTCGAGTTCCGTTG	Construction of pIAE79
**1026**	GGAAAGATGGATCCCACCACTCGC	
**1027**	CTGATATTGGCGGATCCTAATGGC	
**1028**	CGCTTACTACCTGCTCGAGAATATCC	
**1223**	CCTGTATCTGTTAAGAAAGCG	RT-PCR assays for *glnA*
**1224**	CCTTATTTGGCATTTGCTGCC	
**1225**	CTAAAGAACCAGGTGTGGAAGG	
**1207**	TTAAACGTCGTAATAGATAGAGAAC	RT-PCR assays for as_*glnA*
**1222**	GAACTAGCATTGGAAGCGC	
**1209**	AAAAGAGGAGAGAGTTGGTGG	RT-PCR assays for *rnpB*
**1226**	GAGGTACTGGCTCGGTAAACC	
**1227**	GAGGGCGATTATCTATCTGG	

Restriction sites used for cloning are underlined. Mutations are indicated with bold nucleotides.

REFERENCES CITED

Black, T.A., Cai, Y., Wolk, C.P., 1993. Spatial expression and autoregulation of *hetR*, a gene involved in the control of heterocyst development in *Anabaena*. Mol Microbiol 9, 77–84. https://doi.org/10.1111/j.1365-2958.1993.tb01670.x

Buikema, W.J., Haselkorn, R., 1991. Characterization of a gene controlling heterocyst differentiation in the cyanobacterium *Anabaena* 7120. Genes Dev 5, 321–330. https://doi.org/10.1101/gad.5.2.321

Elhai, J., Wolk, C.P., 1988. A versatile class of positive-selection vectors based on the nonviability of palindrome-containing plasmids that allows cloning into long polylinkers. Gene 68, 119–138. https://doi.org/10.1016/0378-1119(88)90605-1

Frías, J.E., Flores, E., Herrero, A., 2000. Activation of the *Anabaena nir* operon promoter requires both NtcA (CAP family) and NtcB (LysR family) transcription factors. Mol Microbiol 38, 613–625. https://doi.org/10.1046/j.1365-2958.2000.02156.x

Frías, J.E., Flores, E., Herrero, A., 1994. Requirement of the regulatory protein NtcA for the expression of nitrogen assimilation and heterocyst development genes in the cyanobacterium *Anabaena* sp. PCC 7120. Mol Microbiol 14, 823–832. https://doi.org/10.1111/j.1365-2958.1994.tb01318.x

Hanahan, D., 1983. Studies on transformation of *Escherichia coli* with plasmids. J Mol Biol 166, 557–580. https://doi.org/10.1016/s0022-2836(83)80284-8

Olmedo-Verd, E., Brenes-Álvarez, M., Vioque, A., Muro-Pastor, A.M., 2019. A heterocyst-specific antisense RNA contributes to metabolic reprogramming in *Nostoc* sp. PCC 7120. Plant Cell Physiol 60, 1646–1655. https://doi.org/10.1093/pcp/pcz087

Shih, P.M., Wu, D., Latifi, A., Axen, S.D., Fewer, D.P., Talla, E., Calteau, A., Cai, F., Tandeau de Marsac, N., Rippka, R., Herdman, M., Sivonen, K., Coursin, T., Laurent, T., Goodwin, L., Nolan, M., Davenport, K.W., Han, C.S., Rubin, E.M., Eisen, J.A., Woyke, T., Gugger, M., Kerfeld, C.A., 2013. Improving the coverage of the cyanobacterial phylum using diversity-driven genome sequencing. Proc Natl Acad Sci U S A 110, 1053–1058. https://doi.org/10.1073/pnas.1217107110
