# Supplementary material for: Antisense RNA regulates glutamine synthetase in a heterocyst-forming cyanobacterium
Source: Plant Physiol. 2024 May 6;195(4):2911–20. doi: 10.1093/plphys/kiae263 (PMC11288750; doi:10.1093/plphys/kiae263)
Supplement: kiae263_Supplementary_Data [file pp.00434.2024-s03.pdf › pp.00434.2024-s03.pdf]

Plant Physiology®

Antisense regulation of glutamine synthetase in a heterocyst-forming cyanobacterium

Isidro Álvarez-Escribano, Belén Suarez-Murillo, Manuel Brenes-Alvarez, Agustin Vioque, and Alicia Muro-Pastor

Review Timeline:

Submission Date:	19-Mar-2024
Editorial Decision:	14-Apr-2024
Revision Received:	19-Apr-2024
Accepted:	19-Apr-2024

14-Apr-2024

Dr. Alicia M Muro-Pastor
Consejo Superior de Investigaciones Cientificas
IBVF
Sevilla
Spain

RE: Antisense regulation of glutamine synthetase in a heterocyst-forming cyanobacterium

Dear Dr. Alicia Muro-Pastor:

We are pleased to accept your manuscript for publication in the Plant Physiology. This acceptance is contingent on revision based on the comments of our reviewers. Following consultation, both reviewers have requested the inclusion of more experimental details for Figure 2 (as outlined below), and further clarification in the text where regulation mechanisms (e.g. post-transcriptional and post-translational) are referred to.

Please highlight all changes and include a detailed annotation to changes to the text, with line numbers, and noting your responses to the comments.

To submit your revised manuscript, click:

Link Not Available

If you cannot return the revised manuscript within 8 weeks of receipt, please let us know. Otherwise, we will assume that you have elected not to revise the manuscript and are withdrawing it.

Thank you for allowing us to review your work. We look forward to hearing from you soon.

Sincerely,

Plant Physiology Board of Editors

-- Reviewer comments:
Reviewer #1 (Comments for the Author):

The glutamine synthetase (GS) enzyme plays a pivotal role in nitrogen metabolism across bacteria, cyanobacteria, and plants, facilitating the transfer of ammonia to glutamate at the expense of ATP. Its activity is tightly regulated through various mechanisms, including transcriptional and posttranslational processes. Notably, in cyanobacteria, GS regulation also involves interaction with small regulatory proteins known as IFs.

In a recent study led by Muro-Pastor, a novel regulatory mechanism of GS expression and activity has been elucidated. This mechanism involves the presence of inhibitory antisense RNA, encoded within the 3' untranslated region (UTR) of the gene adjacent to glnA (which encodes GS), termed the GS inhibitory factor (IF7).

The study employed state-of-the-art molecular techniques to investigate this phenomenon. Initially, the group demonstrated an increase in the 3'UTR of gifA, corresponding to as_glnA, under ammonia-rich conditions. Subsequently, through strategic directed mutagenesis, they generated mutant strains lacking as_glnA and observed alterations in glnA expression and GS activity. Remarkably, under ammonia supply, where GS activity is typically low in wild-type strains, this activity further decreased in the mutant strain.

Further experiments involved overexpression of the gifA coding sequence, including the antisense RNA, which confirmed the downregulation of GS activity by as_glnA. Additionally, a strain carrying a vector encoding the antisense RNA in trans was constructed, demonstrating inhibition of glnA expression by the provided antisense RNA.

In the discussion, the authors provide insights into the genomic arrangement of glnA and gifA in a tail-to-tail orientation and show with a model in figure 5 why members of Nostocales exhibit this organization and inhibition mechanism compared to the smaller cells of unicellular cyanobacterium Synechocystis. Moreover, the study highlights another instance of RNA-RNA interaction-mediated regulation in Nostoc. Finally, the potential for pathway engineering in Nostoc cells, leveraged as cellular factories for bioproducts, is suggested based on these novel findings.

The paper stands out for its clarity in both writing and experimental design, with each step of the research meticulously described and the results presented in a transparent and reproducible manner. The novel findings hold significance beyond the immediate field, appealing to a wider audience interested in metabolic control, gene regulation, and nitrogen metabolism.

The authors' ability to communicate their work effectively makes the paper accessible to researchers from various disciplines, enabling a broader understanding and potential application of the findings. Overall, the paper contributes valuable insights to the scientific community and is poised to generate interest and further exploration in related areas of study.

Reviewer #2 (Comments for the Author):

The manuscript provides evidence for a novel and interesting mode of regulation of expression of the glnA gene in Anabaena based on an overlapping antisense transcript. The gene is critical for cyanobacterial physiology, and the work has wider significance because previous analysis of the Anabaena transcriptome raises the possibility that many other genes may be regulated similarly. The work is very well conducted and I have only minor comments that can be addressed by clearer explanations in the text.

1. Abstract line 59. GS regulation is clearly complex and can operate at many levels: transcriptional, post-transcriptional,. translational, post-translational. I think that makes it really important to be precise in terminology. The statement that "the amount of GS can be modulated" is making an unsubstantiated claim because the authors don't actually measure the amount of GS protein: they measure glnA mRNA levels and they measure GS activity (in Fig 2C). Neither measure necessarily reflects the level of GS protein. "...the amount of GS activity..." would be much better.
2. Line 72, and many later instances. In many places in the text the authors divide regulation mechanisms into "transcriptional" and "post-transcriptional". This use of "post-transcriptional" covers a lot of things - it could be regulation of mRNA stability, it could be regulation of translation or it could be regulation of the stability or activity of the translated protein. In this instance, what the authors actually mean by "post-transcriptional" is post-translational regulation of GS activity. Similarly with line 79 etc etc. I think the authors could make their text much clearer throughout if they specify exactly which level of regulation they are talking about.
3. Similarly, Fig. 5 divides regulation just into transcriptional and post-transcriptional. I think it would help to specify more of the possible levels of regulation.
4. Fig. 2C, Methods lines 440-443, Results lines 171-182. Please specify the basis for comparison of GS activity ie how did you determine the amount of material to go into each measurement? From looking at the paper cited I suspect that samples were standardised according to the wet weight of cell pellets, but please explain it here and give the experimental details.

PP2024-RA-00434

The following is a point-by-point response to the comments by both reviewers:

Reviewer #1 (Comments for the Author):

The glutamine synthetase (GS) enzyme plays a pivotal role in nitrogen metabolism across bacteria, cyanobacteria, and plants, facilitating the transfer of ammonia to glutamate at the expense of ATP. Its activity is tightly regulated through various mechanisms, including transcriptional and posttranslational processes. Notably, in cyanobacteria, GS regulation also involves interaction with small regulatory proteins known as IFs.
In a recent study led by Muro-Pastor, a novel regulatory mechanism of GS expression and activity has been elucidated. This mechanism involves the presence of inhibitory antisense RNA, encoded within the 3' untranslated region (UTR) of the gene adjacent to glnA (which encodes GS), termed the GS inhibitory factor (IF7).

The study employed state-of-the-art molecular techniques to investigate this phenomenon. Initially, the group demonstrated an increase in the 3'UTR of gifA, corresponding to as_glnA, under ammonia-rich conditions. Subsequently, through strategic directed mutagenesis, they generated mutant strains lacking as_glnA and observed alterations in glnA expression and GS activity. Remarkably, under ammonia supply, where GS activity is typically low in wild-type strains, this activity further decreased in the mutant strain.
Further experiments involved overexpression of the gifA coding sequence, including the antisense RNA, which confirmed the downregulation of GS activity by as_glnA. Additionally, a strain carrying a vector encoding the antisense RNA in trans was constructed, demonstrating inhibition of glnA expression by the provided antisense RNA.

In the discussion, the authors provide insights into the genomic arrangement of glnA and gifA in a tail-to-tail orientation and show with a model in figure 5 why members of Nostocales exhibit this organization and inhibition mechanism compared to the smaller cells of unicellular cyanobacterium Synechocystis. Moreover, the study highlights another instance of RNA-RNA interaction-mediated regulation in Nostoc. Finally, the potential for pathway engineering in Nostoc cells, leveraged as cellular factories for bioproducts, is suggested based on these novel findings.

The paper stands out for its clarity in both writing and experimental design, with each step of the research meticulously described and the results presented in a transparent and reproducible manner. The novel findings hold significance beyond the immediate field, appealing to a wider audience interested in metabolic control, gene regulation, and nitrogen metabolism.
The authors' ability to communicate their work effectively makes the paper accessible to researchers from various disciplines, enabling a broader understanding and potential application of the findings. Overall, the paper

contributes valuable insights to the scientific community and is poised to generate interest and further exploration in related areas of study.

THANK YOU!

Reviewer #2 (Comments for the Author):

Please note that line numbers refer to the version with marked changes

The manuscript provides evidence for a novel and interesting mode of regulation of expression of the glnA gene in Anabaena based on an overlapping antisense transcript. The gene is critical for cyanobacterial physiology, and the work has wider significance because previous analysis of the Anabaena transcriptome raises the possibility that many other genes may be regulated similarly. The work is very well conducted and I have only minor comments that can be addressed by clearer explanations in the text.

THANK YOU!

1. Abstract line 59. GS regulation is clearly complex and can operate at many levels: transcriptional, post-transcriptional, translational, post-translational. I think that makes it really important to be precise in terminology. The statement that "the amount of GS can be modulated" is making an unsubstantiated claim because the authors don't actually measure the amount of GS protein: they measure glnA mRNA levels and they measure GS activity (in Fig 2C). Neither measure necessarily reflects the level of GS protein. "...the amount of GS activity..." would be much better.

Line 59: Change introduced as suggested.

2. Line 72, and many later instances. In many places in the text the authors divide regulation mechanisms into "transcriptional" and "post-transcriptional". This use of "post-transcriptional" covers a lot of things - it could be regulation of mRNA stability, it could be regulation of translation or it could be regulation of the stability or activity of the translated protein. In this instance, what the authors actually mean by "post-transcriptional" is post-translational regulation of GS activity. Similarly with line 79 etc etc. I think the authors could make their text much clearer throughout if they specify exactly which level of regulation they are talking about.

We have replaced post-transcriptional with post-translational in several instances (lines 51, 73, 80). However, when the possible mechanism(s) we refer to can be either post-transcriptional or post-translational (or both) we use post-transcriptional to cover both possibilities (lines 117, 290).

3. Similarly, Fig. 5 divides regulation just into transcriptional and post-transcriptional. I think it would help to specify more of the possible levels of regulation.

Figure 5 has been re-drawn to include three levels of regulation (transcriptional, post-transcriptional and post-translational) as suggested by the reviewer. These

three levels are also mentioned in the text (lines 304-305). The legend to Figure 5 (lines 573-584) has been rewritten accordingly.

4. Fig. 2C, Methods lines 440-443, Results lines 171-182. Please specify the basis for comparison of GS activity ie how did you determine the amount of material to go into each measurement? From looking at the paper cited I suspect that samples were standardised according to the wet weight of cell pellets, but please explain it here and give the experimental details.

The methods section specifies now that GS activity was normalized according to the chlorophyll content of the cell sample used (lines 453-454). Legend to Figure 2 (line 540) and the result section (lines 179-182) also include now the normalization used.

19-Apr-2024
Dr. Alicia M Muro-Pastor
Consejo Superior de Investigaciones Cientificas
IBVF
Sevilla
Spain

MSID: PP2024-RA-00434R1
MS TITLE: Antisense regulation of glutamine synthetase in a heterocyst-forming cyanobacterium

Dear Dr. Alicia Muro-Pastor:

I am pleased to inform you that your revised manuscript "Antisense regulation of glutamine synthetase in a heterocyst-forming cyanobacterium" has been accepted for publication in Plant Physiology. Your manuscript will be checked for consistency with journal formatting and image standards by one of our Science Editors, who will contact you shortly. They may have a number of queries and suggestions for you. Please keep in mind that they work to increase the online visibility of your article and its attraction for readers from the widest of backgrounds. You will have the opportunity to review any revisions, and I hope you will work constructively with them to get the most out of your research. You will also have the opportunity to review all changes when you receive the author proofs for your article.

Once validated for publication, your article will be sent to a Science Editor for a final review of your manuscript and source files. After your final files are uploaded, your article will appear in the next available issue and the online Preview version of your article will be posted after your signed Copyright and Checklist forms and final source files are received.

Please be sure to include the following in your manuscript file:
Harvard style citations
List of all author contributions and funding information at the beginning of manuscript after the titles and author list.
List all supplemental materials associated with the article, with titles, at the end of the ms, before the references and acknowledgments section.
Complete Figure Legends for all figures appearing in the article listed before the Literature Cited.

Thank you and congratulations on your Plant Physiology paper!

Sincerely,
Plant Physiology

=====
FIRST AUTHOR PROFILES
=====

We recognize the hard work that goes into being First Author of a Plant Physiology paper by publishing a "First Author Feature" for each First Author alongside your paper.

If the first author(s) would like to submit information for a feature on Facebook and Twitter, fill out the form by clicking on the link below:

Plant Physiology First Author Feature Form: https://forms.office.com/r/ZBCGZfhm8F
If you cannot access the form through the link above, please contact Rachel at rbelsky@aspb.org.

To upload an image of yourself to accompany your profile, please put your photo in the correct journal folder at the link below. You must label your image: Author First Name Last Name Article Title.

First Author Profile Image Dropbox: https://www.dropbox.com/scl/fo/xz029p7qjr2jml2rp2kmq/h?dl=0&rlkey=6fkxcaqmxx35ap152b2osy1fz

=====

IMPORTANT REMINDER: PEER REVIEW REPORTS
=====

If you opted to publish a peer review report along with your article during the original submission process, it will be prepared by the editorial staff and publicly posted with your manuscript, inside the zip file that contains any other supplemental material. As a reminder, the peer review report is a public record of all comments from editors and reviewers, as well as your prior responses, as you received them in the decision letters for each draft of your manuscript. If you agreed to publish this report and have changed your mind, or are not sure if you selected this option, please contact the editorial office as soon as possible before signing the license agreement from our publisher.

-- Reviewer comments:
